# Univariable and multivariable Mendelian randomization investigating the effects of telomere length on the risk of adverse pregnancy outcomes

**DOI:** 10.3389/fendo.2023.1225600

**Published:** 2023-08-03

**Authors:** Xinyu Han, Tianqiang Wu, Chun yan Liu

**Affiliations:** ^1^ Department of First Clinical Medical College, Heilongjiang University of Chinese Medicine, Harbin, China; ^2^ Department of Endocrinology, The First Affiliated Hospital of Heilongjiang University of Chinese Medicine, Harbin, China

**Keywords:** Mendelian randomization, telomere length, spontaneous abortion, preterm birth, adverse pregnancy outcomes, causality

## Abstract

**Background:**

Numerous observational studies have revealed a correlation between telomere length (TL) and adverse pregnancy outcomes (APOs). However, the impacts of TL on APOs are still unclear.

**Methods:**

Mendelian randomization (MR) was carried out using summary data from genome-wide association studies (GWAS). Inverse variance weighted (IVW) was employed as the primary analysis to explore the causal relationship between TL and APOs. The exposure data came from a GWAS dataset of IEU analysis of the United Kingdom Biobank phenotypes consisting of 472,174 European participants. Summary-level data for five APOs were obtained from the GWAS datasets of the FinnGen consortium. We also performed multivariate MR (MVMR), adjusting for smoking, alcohol intake, body mass index (BMI), and number of live births. In addition, we conducted a series of rigorous analyses to further examine the validity of our MR findings.

**Results:**

After Bonferroni correction and rigorous quality control, univariable MR (UVMR) demonstrated that a shorter TL was significantly associated with an increased risk of spontaneous abortion (SA) (odds ratio [OR]: 0.815; 95% confidence interval [CI]: 0.714–0.930; *P* = 0.002) and preterm birth (PTB) (OR: 0.758; 95% CI: 0.632-0.908; *P* = 0.003) in the IVW model. There was a nominally significant relationship between TL and preeclampsia (PE) in the IVW model (OR: 0.799; 95% CI: 0.651-0.979; *P* = 0.031). However, no significant association was found between TL and gestational diabetes mellitus (GDM) (OR: 0.950; 95% CI: 0.804-1.122; *P* = 0.543) or fetal growth restriction (FGR) (OR: 1.187; 95% CI: 0.901-1.565; *P* = 0.223) among the five statistical models. Furthermore, we did not find a significant causal effect of APOs on TL in the reverse MR analysis. MVMR analysis showed that the causal effects of TL on SA remained significant after accounting for smoking, alcohol intake, BMI, and number of live births.

**Conclusion:**

Our MR study provides robust evidence that shorter telomeres were associated with an increased risk of SA. Further work is necessary to investigate the potential mechanisms. UVMR and MVMR findings showed limited evidence that TL affects the risk of PTB, PE, GDM, and FGR, illustrating that the outcomes of previous observational studies may have been confounded.

## 1 Introduction

Located at the extremities of chromosomes, telomeres are DNA-protein complexes that safeguard genomic stability and integrity by shielding chromosome ends from erosion and fusion (1). With each cell division, telomeres become shorter, which is regarded as an indicator of cellular aging (2). Multiple prior investigations have shown that genetics is the primary determinant of TL (3, 4). Telomere attrition can cause DNA damage, induce cellular senescence and apoptosis, and is a well-known risk factor for a variety of age-related disorders, such as premature ovarian failure, infertility (5), diabetes (6), cardiovascular diseases (7), and neurodegenerative diseases (8). APOs include SA, PTB, PE, GDM, and FGR, which pose a grave threat to maternal, fetal, and neonatal health and significantly cause maternal and infant mortality (9). Given their negative immediate and long-term effects, it is imperative to address APOs. The shortening of telomeres, which can influence the aging of placental and fetal membrane cells, has been associated with aberrant placental aging and APOs (10). Increasing evidence indicates that TL in placentas or fetal membrane may function as a potential biomarker for predicting APOs (11). The length of telomeres can be measured in numerous types of tissue, with minor variations depending on the cell type. Currently, peripheral blood leukocyte TL is the most common type in TL research, possibly due to the convenience of obtaining blood samples. In addition, a recent study evaluated the feasibility of using peripheral blood leukocyte TL to represent other types of tissue TL by detecting the relative telomere length (RTL) of various types of tissues from 952 Genotype-Tissue Expression (GTEx) donors. In 23 categories of tissues, whole blood RTL correlated positively with 15 tissue-specific RTL measurements, demonstrating that peripheral blood leukocyte RTL can represent RTL in the majority of tissues (12). Despite the fact that no study has directly confirmed the correlation between peripheral blood leukocyte TL and placental TL, some studies have found a positive correlation between the TERC mRNA level in term placenta of PE patients at 36-38 weeks and the TERC mRNA level in their peripheral blood Monocyte PBMC (r=0.21, p=0.024) (13). TERC is a gene for telomere maintenance that restores telomerase activity and prolongs cell life. Insufficient expression of this gene can contribute to telomeric lesions, accelerate the accumulation of aging cells, and reduce TL (14). The article examines the possible correlation between TL in peripheral blood and placenta through an indirect approach. Two additional studies also imply the synchronicity of TL in peripheral blood and fetal membranes in an indirect way. Alrefaei et al. discovered that the telomere length of human fetal mesenchymal stem cells (hFM MSCs) in mothers aged 30-39 and 40 was substantially shorter compared to that of young mothers aged 20-29 (15). A study conducted by Nsereko et al. on 297 expectant women from Rwanda revealed a significant negative correlation between maternal age and the TL of peripheral blood leukocytes, consistent with the findings of studies on fetal membrane TL (16). Moreover, similar to TL of placental or fetal membrane, multiple studies have also found an association between maternal peripheral blood leukocyte TL and APOs. A case-control study revealed that couples with idiopathic recurrent pregnancy loss (iRPL) had substantially shorter peripheral blood leukocyte TL than healthy control couples (17). Another prospective study involving 100 expectant Mexican women found that peripheral blood TL during late gestation was substantially lower in women with PTB compared to those with full-term delivery (*P*=0.02), supporting the association between maternal TL and the incidence of PTB (18). In a case-control study comparing relative TL of genomic DNA extracted from peripheral blood leukocytes in 113 GDM patients and 396 normal pregnant women, GDM patients demonstrated significantly shorter RTL than normal controls (*P* = 0.046), suggesting that TL is negatively correlated with the incidence of GDM (19). However, a study involving 1,228 women of reproductive age attempting natural conception with a history of 1-2 pregnancy losses revealed that preconception leukocyte TL is unrelated to fecundability, pregnancy loss, or live birth (20). This inconsistency in observational study findings can be attributed to sample size bias and residual confounders. Therefore, the causal relationships between TL and APOs remain ambiguous and require more convincing evidence for validation. Using genetic variations that are closely linked to exposure as instrumental variables (IVs), MR is a reliable method for determining the causality between exposure and outcome (21). Similar to a randomized controlled trial, the MR design is less susceptible to bias from confounding factors and reverse causality due to the random assignment of genetic variants at conception (22, 23). MVMR is an emerging technique that allows simultaneous assessment of relevant exposures by incorporating genetic variants of multiple risk factors into the same model to minimize the impact of confounding variables (24). Therefore, UVMR and MVMR analyses were conducted to ascertain the causal relationships between TL and the five prevalent APOs.

## 2 Materials and methods

### 2.1 Study design

**Figure 1:**
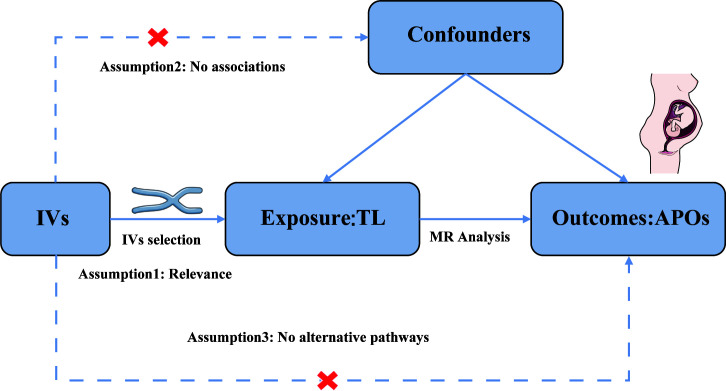
Assumptions of the Mendelian randomization (MR) analysis for TL and the risk of APOs.

We performed a two-sample MR analysis to assess the causal relationship of TL with APOs using publicly available data. **Figure 1** illustrates the study’s methodology. The genetic variants selected to estimate the causal effect must satisfy three key assumptions (25): Assumption 1, genetic variants should be strongly associated with TL (*P* < 5 × 10-8); Assumption 2, The association between genetic variants of TL and APOs is independent of confounding factors; Assumption 3, Genetic variants affect the risk of APOs directly through TL, not through other pathways. Evidence from previous observational clinical trials and MR studies indicated that smoking, alcohol intake, BMI, and number of live births are risk factors for the development of APOs (26–31). Therefore, we further conducted MVMR adjusting for genetic liability to smoking, alcohol intake, BMI, and number of live births. Since our data were derived from publicly accessible GWAS summary statistics, no ethical approval was necessary.

### 2.2 Data sources

The summary statistics for TL were derived from the largest GWAS on TL to date (dataset ID: ieu-b-4879), which included 472,174 European participants from the UK Biobank (UKB), with a comparable distribution of males (45.8%) and females (54.2%) (32). Quantitative polymerase chain reaction (PCR) analyses were performed to obtain Leukocyte TL measurements for these participants from the UKB (33). GWAS summary data for SA (9,113 cases and 89,340 controls), PTB (5,480 cases and 98,626 controls), PE (3,903 cases and 114,735 controls), GDM (5,687 cases and 123,579 controls), and FGR (2,579 cases and 171,167 controls) were obtained from the FinnGen consortium (34). All participants in this study are of European descent. In addition, we gained aggregate data regarding smoking, alcohol intake, BMI, and number of live births from the Neale Lab or MRC-IEU consortium. All GWAS data samples originate from the European populations. **Table 1** provides a summary of all datasets included in this investigation.

**Table 1:** Details of studies included in Mendelian randomization (MR) analyses.

Traits	Data source	Author and year	Sample size(cases/controls)	Sex	Number of SNPs	Ancestry	GWAS ID
Exposure							
TL	UK Biobank	Codd et al. (2021)	472,174	Males and Females	20,134,421	European	ieu-b-4879
Smoking	Neale Lab	Neale et al. (2017)	337,030	Males and Females	10,894,596	European	ukb-a-16
Alcohol intake	Neale Lab	Neale et al. (2017)	336,965	Males and Females	10,894,596	European	ukb-a-25
Body mass index	Neale Lab	Neale et al. (2017)	336,107	Males and Females	10,894,596	European	ukb-a-248
Number of live births	MRC-IEU	Ben Elsworth et al. (2018)	250,782	Males and Females	9,851,867	European	ukb-b-1209
Outcomes							
SA	FinnGen	NA. (2021)	9,113/89,340	Males and Females	16,379,138	European	finn-b-O15_ABORT_SPONTAN
PTB	FinnGen	NA. (2021)	5,480/98,626	Males and Females	16,379,340	European	finn-b-O15_PRETERM
PE	FinnGen	NA. (2021)	3,903/114,735	Males and Females	16,379,723	European	finn-b-O15_PRE_OR_ECLAMPSIA
GDM	FinnGen	NA. (2021)	5,687/123,579	Males and Females	16,379,784	European	finn-b-GEST_DIABETES
FGR	FinnGen	NA. (2021)	2,579/171,167	Males and Females	16,382,867	European	finn-b-O15_POOR_FETGRO

TL, telomere length; SA, spontaneous abortion; PTB, preterm birth; PE, preeclampsia; GDM, gestational diabetes mellitus; FGR, fetal growth restriction.

### 2.3 Selection and evaluation of instrumental variable

To satisfy the three assumptions of the MR analysis, we selected the IVs through the following procedure. Initially, all SNPs substantially associated with TL (*P* < 5 × 10 ^-8^) were chosen as IVs. When SNPs that strongly predicted APOs were extracted at the genome-wide significance level (*P* < 5 × 10 ^-8^), the number of available SNPs was low or even absent, consequently, a cut off (*P* < 5 × 10 ^-6^) was adopted to obtain SNPs that predicting APOs in the reverse MR analysis. The corresponding linkage disequilibrium was then evaluated to confirm that there were SNPs in a linkage disequilibrium state and that the SNPs were independent by removing SNPs from a 10,000-kb window with an r^2^ < 0.001 thresholds. Thirdly, putative pleiotropic effects were eliminated by retrieving the secondary phenotype of each SNP from PhenoScan V2 (35). SNPs corresponding to the phenotypes associated with the results were excluded from further analysis, while the remaining SNPs were utilized.

Variance (R2) and F-statistic were employed to assess the robustness of IVs to avoid weak tool bias. We calculated the F-statistic for each SNP by adopting the formula: F = R^2^/(1-R^2^) [(N-K-1)/K], where N is the sample size, k is the total number of SNPs selected for MR analysis, and R^2^ is the total proportion of phenotypic variations explained by all the SNPs in our MR model (36). The following formula was used to calculate the R^2^ for each SNP: R^2^ = Σ [2 × (1 – MAF) × MAF × β^2^/(SE^2^ × N)], where SE and β are the standard error and β coefficient for effect size, MAF is the minor allele frequency for each SNP (36). An F-statistic larger than 10 was deemed significant enough for the association between IVs and exposure to prevent the MR analyses from being influenced by weak tool bias (25). Statistical power for each outcome was calculated by utilizing the online tool (https://shiny.cnsgenomics.com/mRnd/) (37). A sufficient power of over 80% was recommended.

### 2.4 Statistical analysis

The IVW method was employed as the primary analytical method for estimating causal effects between TL and APOs. Additional analysis methodologies included MR-Egger, weighted median, weighted mode, and simple mode. The IVW method is an extension of the Wald ratio estimator based on meta-analytic principles that can provide an accurate estimate in an ideal state where all included SNPs are presumed to be valid IVs without pleiotropy (38). MR-Egger permits certain SNPs to influence the outcome through mechanisms other than exposure modification (39). In addition, the MR-Egger intercept can detect and adjust pleiotropy (39). To employ the weighted median method, at least 50 percent of intravenous solutions must satisfy the assumption that they are valid intravenous solutions (40). The weighted model method clusters SNPs and calculates estimates based on the cluster containing the greatest number of SNPs (39). Lastly, even though the simple model method is less effective than IVW, it provides robustness for pleiotropy (41). Based on prior research (26–31), we adjusted for smoking, alcohol intake, BMI, and number of live births in multivariate MR to demonstrate a causal effect of TL on APOs. The methods we applied to execute MVMR included IVW and MR-Lasso (42).

Various sensitivity analyses were implemented in this study to ensure the stability and reliability of the MR results. First, Cochran’s Q test was applied to evaluate the heterogeneity between SNPs, where a *p*-value greater than 0.05 indicated no heterogeneity. Secondly, the MR-Egger intercept was utilized to quantify the horizontal pleiotropy of IVs. Thirdly, we performed the leave‐one‐out analysis to check whether the MR results were driven by any single SNP. Finally, we conducted the MR-PRESSO to detect potential outlier SNPs (42). A two-sided *p*-value < 0.05 was considered statistically significant. We modified the *p*-value by Bonferroni correction for the number of outcomes. For the primary analysis (association of TL with five APOs), the association with a two-sided *p*-value < 0.01 (where α = 0.05/5 outcomes) was deemed statistically significant and a two-sided *p*-value < 0.05 was thought suggestive. MR analyses were conducted using the TwoSampleMR (version 0.5.6) and MVMR (version 0.3) in R. All data analyses were performed with R version 4.2.2.

## 3 Result

### 3.1 Genetic instruments

In the present study, 117, 107, 109, 108, and 93 SNPs were eventually obtained as the IVs for TL to assess the associations between TL and SA, PTB, PE, GDM, and FGR, respectively **(** **Supplementary Tables S1**–**S5** **)**. The F statistic for all these genetic variants was above the threshold of 100, indicating a low likelihood of weak instrumental bias.

The statistical power of SA, PTB, and PE was above 80 percent, which is sufficient to prove the reliability of the results. However, the statistical power of GDM and FGR is less than 80%, which may lead to false negatives. The detailed results of the selected IV’s strength and statistical power are shown in **Table 2**. For the MR analysis of APOs on TL, 15, 9, 7, 10, and 10 SNPs associated with SA, PTB, PE, GDM, and FGR were selected respectively **(** **Supplementary Tables S6**–**S10** **)**. However, the F statistic of most IVs was less than 10, suggesting the possibility of weak instrumental bias.

**Table 2:** The results of the selected IVs strength and statistical power.

Outcomes	R^2^ for TL (Total)	F for TL (Total)	Power
SA	0.028	114.517	0.86
PTB	0.023	103.153	0.80
PE	0.029	129.184	0.99
GDM	0.029	129.969	0.24
FGR	0.020	104.329	0.27

SA, spontaneous abortion; PTB, preterm birth; PE, preeclampsia; GDM, gestational diabetes mellitus; FGR, fetal growth restriction.

### 3.2 Estimated causal effect of TL on APOs

**Figure 2:**
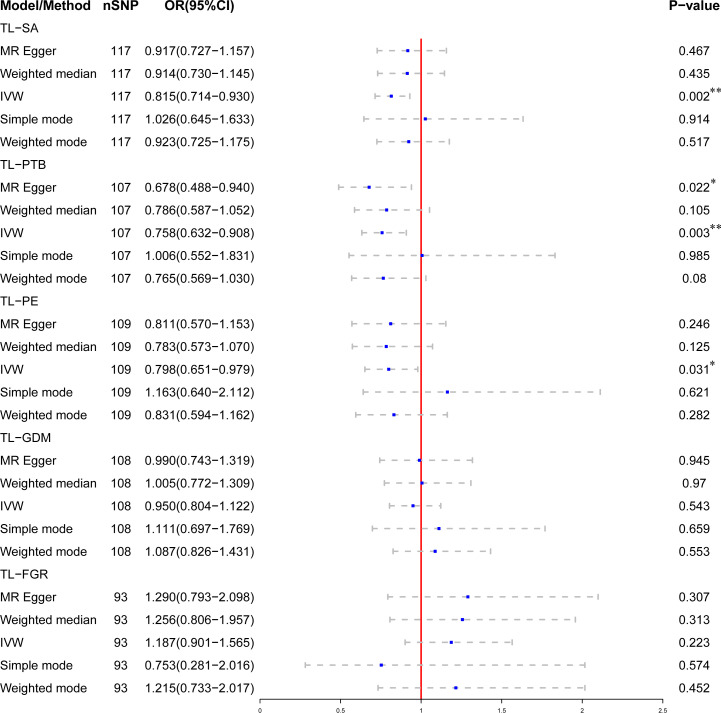
Association of genetically predicted telomere length (TL) and the risk of adverse pregnancy outcomes (APOs). *Indicates that the relationship has nominal statistical significance; **Indicates that the *P*-value meets the Bonferroni correction threshold.

**Figure 3:**
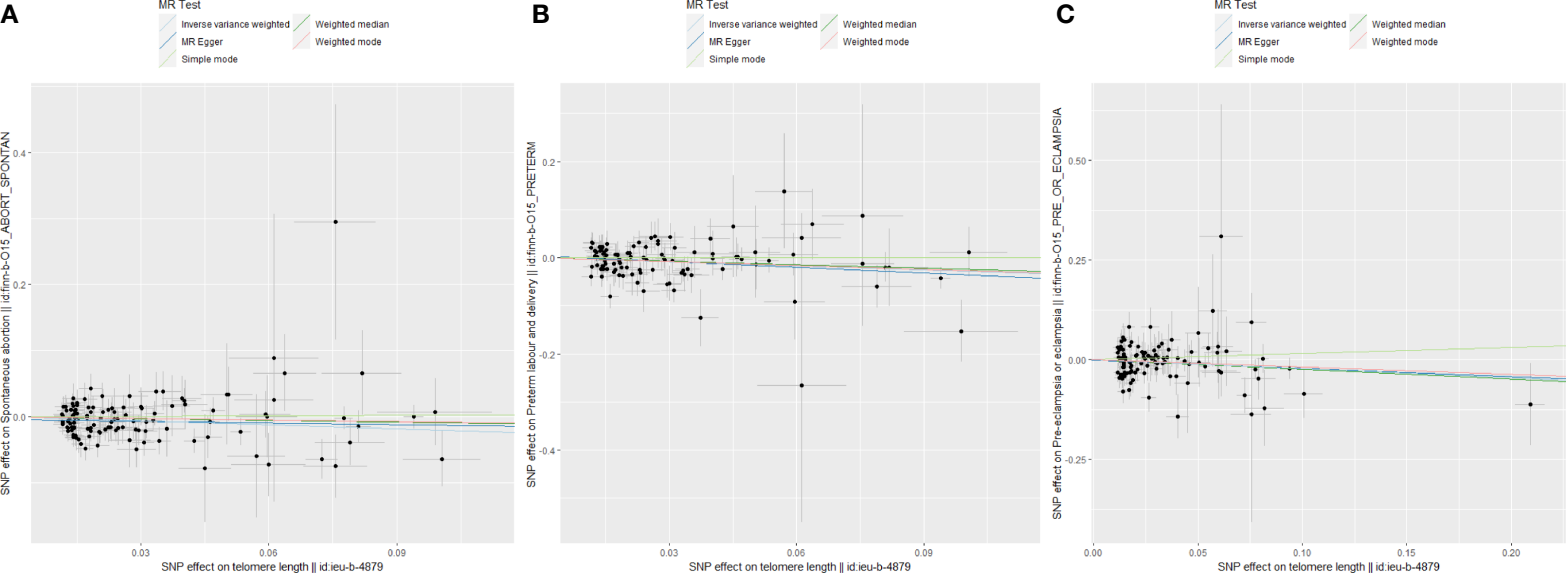
Scatter plots for Mendelian randomization (MR) analyses of the correlation between telomere length (TL) and adverse pregnancy outcomes (APOs). **(A)** SA; **(B)** PE; **(C)** PTB.

**Figure 4:**
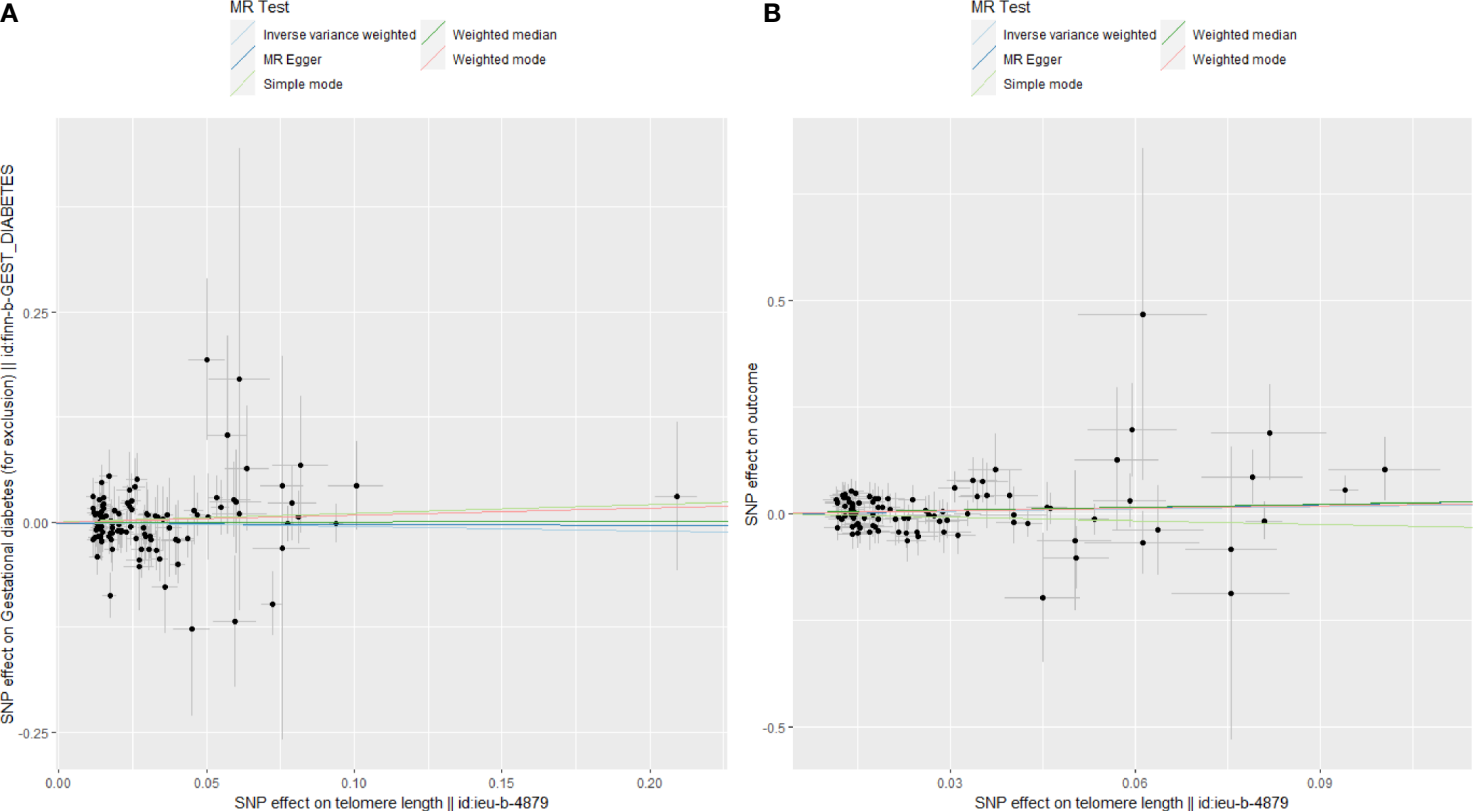
Scatter plots for Mendelian randomization (MR) analyses of the correlation between telomere length (TL) and adverse pregnancy outcomes (APOs). **(A)** GDM; **(B)** FGR.

After Bonferroni correction, we observed that a shorter TL was significantly associated with a higher risk of SA (OR: 0.815; 95%CI: 0.714-0.930; *P* = 0.002) and PTB (OR: 0.758; 95% CI: 0.632-0.908; *P* = 0.003) in IVW model. There was a nominally significant relationship between TL and PE in the IVW model (OR: 0.799; 95% CI: 0.651-0.979; *P* = 0.031). However, no significant association was found between TL and GDM (OR: 0.950; 95% CI: 0.804-1.122; *P* = 0.543) or FGR (OR: 1.187; 95% CI: 0.901-1.565; *P* = 0.223) among the five statistical models. The causal associations of genetically predicted TL and the risk of APOs are presented in **Figure 2**. The scatter plots of the association between TL and APOs are shown in **Figures 3**, **4**. The Cochran’s Q test showed no heterogeneity, and the MR-Egger intercept test found no evidence of horizontal pleiotropy in the MR analysis results. Additionally, the MR-PRESSO results showed no outlier SNPs. **Table 3** provides details on the sensitivity analysis results. The leave-one-out plots further support the robustness of our results and suggest that the effects of any single SNP were unlikely to influence causal estimates (**Supplementary Figures S1**–**S5**).

**Table 3:** Heterogeneity, horizontal pleiotropy, and MR-PRESSO tests of the associations between TL and APOs.

Outcomes	Pleiotropy test			Heterogeneity test						MR-PRESSO
	MR-Egger			MR-Egger			Inverse-variance weighted			Global Test
	Intercept	SE	*P*	Q-value	Q-df	Q-pval	Q-value	Q-df	Q-pval	*P*value
SA	-0.004	0.003	0.227	115.222	115	0.477	116.700	116	0.464	0.488
PTB	0.004	0.005	0.424	100.560	105	0.604	101.205	106	0.613	0.287
PE	-0.001	0.005	0.916	117.849	107	0.223	117.861	108	0.243	0.269
GDM	-0.002	0.004	0.727	91.821	106	0.835	91.943	107	0.850	0.580
FGR	-0.003	0.007	0.685	83.659	91	0.695	83.825	92	0.716	0.777

SA, spontaneous abortion; PTB, preterm birth; PE, preeclampsia; GDM, gestational diabetes mellitus; FGR, fetal growth restriction; MR-PRESSO, Mendelian randomization pleiotropy residual sum and outlier; Q-value, the statistics of Cochran's Q test; SE, standard error.

In the multivariable MR analysis, adjusting for smoking, alcohol intake, BMI, and number of live births, the magnitude of associations between genetic liability to TL and SA diminished, whereas the causal relationship between TL and SA (OR: 0.867; 95%CI: 0.763-0.985; P = 0.028) remained significant. After removing heterogeneous SNPs, the results of the MR-Lasso test remained constant. The F-statistics were 42.50, indicating that there is no potential mild instrument bias. The association between TL and PTB or PE did not persist, however, after adjusting for smoking, alcohol intake, BMI, and number of live births. **Table 4** presents the MVMR results in detail.

**Table 4:** Causal estimates of TL on SA, PTB and PE in MVMR.

Method	Outcome	MVMR	Beta	SE	OR	*P*-value
Adjusted for smoking	SA	IVW	-0.128	0.065	0.879(0.774-0.998)	0.046*
		MR-Lasso	-0.138	0.063	0.871(0.769-0.985)	0.028*
Adjusted for alcohol	SA	IVW	-0.145	0.066	0.865 (0.759- 0.985)	0.029*
		MR-Lasso	-0.166	0.063	0.847(0.748-0.958)	0.009*
Adjusted for BMI	SA	IVW	-0.147	0.066	0.863(0.759-0.983)	0.026*
		MR-Lasso	-0.156	0.065	0.856(0.753-0.971)	0.016*
Adjusted for number of live births	SA	IVW	-0.126	0.064	0.882(0.778-0.999)	0.051
		MR-Lasso	-0.136	0.063	0.873(0.771-0.988)	0.031*
Adjusted for all	SA	IVW	-0.143	0.065	0.867(0.763-0.985)	0.028*
		MR-Lasso	-0.154	0.065	0.857(0.755-0.974)	0.018*
Adjusted for smoking	PTB	IVW	-0.153	0.080	0.858(0.734-1.004)	0.055
		MR-Lasso	-0.139	0.079	0.870(0.745-1.016)	0.077
Adjusted for alcohol	PTB	IVW	-0.150	0.079	0.861(0.737-1.005)	0.058
		MR-Lasso	-0.154	0.078	0.857(0.736-0.999)	0.048*
Adjusted for BMI	PTB	IVW	-0.179	0.090	0.836(0.701-0.997)	0.047*
		MR-Lasso	-0.168	0.080	0.845(0.723-0.989)	0.035*
Adjusted for number of live births	PTB	IVW	-0.151	0.079	0.860(0.737-1.004)	0.055
		MR-Lasso	-0.131	0.078	0.877(0.753-1.022)	0.094
Adjusted for all	PTB	IVW	-0.173	0.089	0.841(0.706-1.001)	0.051
		MR-Lasso	-0.145	0.080	0.865(0.740-1.012)	0.072
Adjusted for smoking	PE	IVW	-0.195	0.110	0.823 (0.663-1.021)	0.076
		MR-Lasso	-0.166	0.094	0.847(0.705-1.018)	0.079
Adjusted for alcohol	PE	IVW	-0.193	0.105	0.824(0.671-1.013)	0.067
		MR-Lasso	-0.161	0.094	0.851(0.708-1.024)	0.087
Adjusted for BMI	PE	IVW	-0.138	0.100	0.871(0.716-1.060)	0.168
		MR-Lasso	-0.093	0.095	0.911(0.756-1.098)	0.325
Adjusted for number of live births	PE	IVW	-0.191	0.109	0.826(0.667-1.023)	0.080
		MR-Lasso	-0.177	0.095	0.838(0.695-1.010)	0.062
Adjusted for all	PE	IVW	-0.131	0.101	0.877(0.330-1.069)	0.196
		MR-Lasso	-0.085	0.095	0.919(0.762-1.106)	0.372

SA, spontaneous abortion; PTB, preterm birth; PE, preeclampsia; MVMR, Multivariable Mendelian randomization; SE, standard error; OR, odds ratio; CI, confidence interval; *indicates that the relationship has nominal statistical significance.

### 3.3 Estimated causal effect of APOs on TL

**Figure 5:**
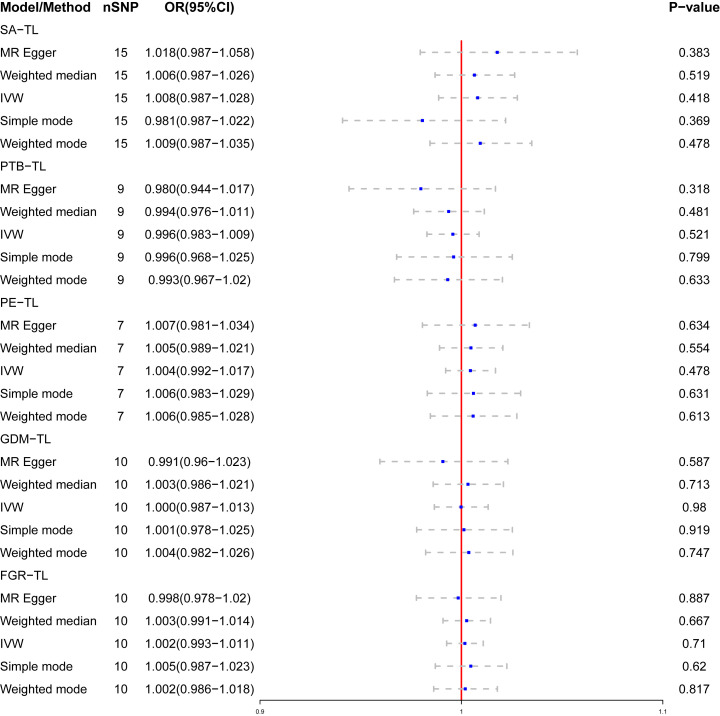
The causal relationship of genetically predicted APOs and TL.

Genetic susceptibility to SA (OR: 1.008; 95% CI: 0.987-1.028, P = 0.418), PTB(OR: 0.996; 95% CI: 0.983-1.009, P = 0.521), PE(OR: 1.004; 95% CI: 0.992-1.017, P = 0.478), GDM(OR: 1.000; 95% CI: 0.987-1.013; P = 0.980), or FGR(OR: 1.002; 95% CI: 0.993-1.011; P = 0.710) was all not causally related to TL in all five statistical models (**Figure 5**). In the sensitivity analyses, no evidence for directional pleiotropy was found when we reanalyzed the five results using MR-Egger regression. Heterogeneity was assessed by Cochrane’s Q test, and the results of IVW and MR Egger analyses revealed heterogeneity only for SA exposure and not for any other APOs. As a result, a random effects model was employed to evaluate the causal relationship between SA and TL. For five APOs, however, no outliers were identified in the MR-PRESSO model. **Table 5** provides details on the sensitivity analysis results for the reverse MR study. In addition, leave-one-out plots demonstrated that individual SNPs were not anticipated to influence causal estimates **(** **Supplementary Figures S6**–**S10** **)**. In addition, scatter plots **(** **Supplementary Figures S11**, **S12** **)** displaying the effect size of each SNP on TL were provided.

**Table 5:** Heterogeneity, horizontal pleiotropy, and MR-PRESSO tests of the associations between APOs and TL.

Exposures	Pleiotropy test			Heterogeneity test							MR-PRESSO
	MR-Egger			MR-Egger			Inverse-variance weighted				Global Test
	Intercept	SE	*P*	Q-value	Q-df	Q-pval	Q-value	Q-df		Q-pval	*P*value
SA	-0.002	0.003	0.574	35.023	13	0.0008	35.919	14		0.001	0.007
PTB	0.002	0.003	0.395	6.476	7	0.485	7.297	8		0.505	0.521
PE	-0.0004	0.002	0.851	2.513	5	0.775	2.552	6		0.863	0.847
GDM	-0.002	0.004	0.727	7.333	8	0.974	7.707	9		0.564	0.606
FGR	0.0009	0.003	0.742	2.206	8	0.695	2.322	9		0.985	0.924

SA, spontaneous abortion; PTB, preterm birth; PE, preeclampsia; GDM, gestational diabetes mellitus; FGR, fetal growth restriction; MR-PRESSO, Mendelian randomization pleiotropy residual sum and outlier; Q-value, the statistics of Cochran's Q test; SE, standard error.

## 4 Discussion

In this investigation, we examined the causal effect of TL on five APOs utilizing MR analyses. We discovered that genetically predicted a shorter TL was associated with an increased risk of certain APOs. The significant correlations were found between TL and SA or PTB After the Bonferroni correction. There was a suggestive association between TL and PE. The casual associations between TL and SA remained significant after controlling for smoking, alcohol intake, BMI, and number of live births. In the MVMR models, the correlation between TL and PTB or PE did not persist.

Our findings that a shortened TL was correlated with an increased risk of SA were consistent with those of prior research. For instance, a recent case-control study found that women with iRPL (n=40) had significantly shorter peripheral blood leukocyte TL in early follicular stages than healthy women (n=41) with a comparable age and body mass index (*P*=0.006) (43). In studies of infertile women undergoing *in vitro* fertilization (IVF), shorter peripheral blood leukocyte TL was linked with a higher incidence of embryonic aneuploidy, a major contributor to pregnancy loss (*P*=0.01) (44). All of these findings support the protective function of a longer TL in SA and suggest that special attention should be paid to patients with a shorter TL along with certain measures should be taken to prevent the development of SA.

A recently published study comparing maternal blood and placental tissue telomere length in PTB and uncomplicated full-term pregnancy (NTP) revealed that maternal blood TL was significantly shorter in PTB (n=11) than in NTP (n=19) and that the proportion of trophoblast cells with shortened telomeres was significantly higher in PTB placental samples (*P*<0.001) (45). In addition, another cohort study found that shorter peripheral blood TL in early pregnancy was significantly associated with an earlier gestational age at delivery (r = 0.35, *P* = 0.02). This association persisted after accounting for maternal age in a linear regression model (OR=0.30, 95% CI: 0.04-0.56, *P*=0.03) (10). Our univariate MR results indicated that shortened TL was significantly associated with the incidence of PTB; however, after adjusting for smoking, alcohol intake, BMI, and number of live births, the correlation became nonsignificant. The discrepancy between the results of MR analysis and observational studies may be due to interference by confounding factors.

Prior investigations have reached contradictory conclusions regarding the relationship between TL and PE, with one study reporting a shorter TL in placenta samples from expectant women with both TL and PE (46). The proportion of trophoblast cells with short TL was significantly higher in early (n=7) and late (n=6) pre-eclamptic placenta samples than in healthy controls (n=13; *P*=0.03), according to a separate study (47). In contrast, a case-control study from China demonstrated that a lengthier leukocyte TL in peripheral blood was associated with PE (48). A study found no statistically significant difference in telomere length between maternal blood and placental samples from patients with PE at 31 to 40 weeks of gestation (n=31) and normal pregnancy controls (n=30) (49). This is consistent with our findings, in which a nominally significant association was found between TL shortening and PE incidence in our univariate MR results, but this relationship became nonsignificant when smoking, alcohol intake, BMI, and number of live births were considered in a multivariable model. These findings suggest that the relationship between TL length and PE is divergent or even contradictory, requiring further investigation.

Our MR investigation identified no correlation between peripheral blood TL and either GDM or FGR. The relative TL of genomic DNA extracted from peripheral blood leukocytes was substantially shorter in patients with GDM than in controls (*p* = 0.046) in a previous case-control study that did not adjust for confounding variables (19). After adjusting for age, another prospective cohort study found no significant correlation between TL and GDM risk (50). This corresponds with our findings. Numerous studies have found a link between shortened placental trophoblast telomere length and FGR (51–53), but none have explored the relation between peripheral blood TL and FGR. Our study found no correlation between peripheral blood TL and FGR. The discrepancy between this result and those of observational studies may be due to the influence of confounding variables on the one hand, and the inability of peripheral blood leukocyte TL to fully reflect placental TL on the other hand, despite the fact that TL is typically consistent across tissues (12). The relationship between leukocyte TL and placental TL and its association with FGR requires additional study.

The results of our reverse MR analysis did not provide genetic evidence of a causal relationship between APOs and TL. There is currently no observational study examining the relationship between APOs and TL; however, studies have found that women with APOs, such as SA, PE, and PTB, have a higher risk of cardiovascular diseases closely related to short telomeres (7, 54, 55), suggesting that APOs may be correlated with TL shortening. This inference is not confirmed by the results of our reverse MR analysis. However, there is a weak instrumental bias in this result, which may contribute to an underestimation of the correlation between exposure and outcome in the two-sample MR study. Insufficient sample size is the primary contributor to this bias. Therefore, we should explain this result with caution and employ larger sample sizes of SA-related GWAS data for verification in the future.

The precise mechanism underlying the connection between TL and APOs is still unknown. Despite this, several studies have pointed out that accelerated TL shortening leads to increased oxidative stress and immune-inflammatory responses, both of which are known to play crucial roles in the development of APOs (56, 57). Telomere attrition may promote placental and fetal membrane cell senescence, which may contribute to the development of APOs (58). Moreover, an earlier study reveals that TL can indicate ovarian reserve function in women. In particular, the age of natural menopause is delayed by approximately 10.2 months for every 1 kilobase (kb) increase in leukocyte TL (59). Reduced ovarian reserve function is a risk factor for miscarriage (60).

The following is a discussion on the potential mechanism of a causal relationship between TL and SA at the genetic level. TL is primarily determined by genes, and previous research has demonstrated that genetic factors can account for 36% to 84% of TL variation (61). Certain gene mutations or functional abnormalities may result in telomere shortening. For example, Poly ADP Ribose Polymerase1 (PARP1), Poly ADP Ribose Polymerase1 (PARP2), and Telomeric Repeat Binding Factor 2 (TERF2/TRF2) (62). PARP1 and PARP2 are ADP ribose transferase (ART) family members. ART is a multifunctional protein Post-translational modification enzyme that participates in numerous cellular processes, such as DNA damage repair, lipid metabolism, immune response, transcriptional regulation, and cell death and plays a crucial role in maintaining gene stability and TL (62). In addition, PARP can prevent telomere degradation and lengthen telomeres by regulating telomerase reverse transcriptase gene transcription (63). Studies have demonstrated that catalytic inhibition or gene deletion of PARP-1 and PARP-2 in the mouse uterus can increase p53 signaling and the population of senescent decidual cells, causing decidualization failure and pregnancy loss in rodents (64). Additionally, PARP-2 was found to be upregulated in the endometrial stromal area (implantation area) during the receptive stage but downregulated during pregnancy failure and pseudopregnancy. Experiments *in vitro* have demonstrated that PARP-2 regulates endometrial receptivity by taking part in cysteine protease 8-dependent inflammation and apoptosis (65). TERF2/TRF2 is a multifunctional telomeric protein that protects the extremities of chromosomes by binding to repetitive sequences on telomere DNA and forming structurally stable telomere caps. The expression of TERF2/TRF2 in the abortion tissue of women with iRPL is substantially reduced compared to that of induced abortion patients, and the TL of iRPL is significantly shorter. This suggests that the downregulation of TERF2/TRF2 expression results in telomere uncapping, which plays a crucial role in pregnancy loss (66). Other studies have demonstrated that TERF2 can regulate autophagy by binding to High Mobility Group Box 1 (HMGB1), a non-histone Chromatin-related protein (67). In recent years, a large number of studies have suggested that abnormalities in autophagy may be associated with SA by modulating decidualization, trophoblast cells, and immune cells at the maternal fetal interface (68). In our study, SNPs used as IVs include rs139795227, rs3093888, and rs3785074, which correspond to the PARP1, PARP2, and TERF2/TRF2 genes, respectively. Therefore, we conclude that genes associated with telomere shortening may be involved in the pathogenesis of SA via inflammatory response, apoptosis, autophagy, and other mechanisms.

According to our knowledge, this is the first time the MR framework was employed for estimating the genetic causality between TL and APOs. This MR study has several strengths that should be noted. First, we eliminated genetic variants linked with potential confounders frequently observed in epidemiological studies and selected only SNPs strongly associated with TL. Second, the large sample size of our MR analysis boosted our statistical power and provided solid evidence of the existence of relationships. Thirdly, we conducted multiple sensitivity analyses to validate the reliability of these results. Lastly, we utilized MVMR to investigate the direct impact of TL on APOs after adjusting for smoking, alcohol intake, BMI, and number of live birth. Despite the benefit, there are some restrictions. First, as a result of using summary-level data from the GWAS database, we were unable to evaluate the nonlinear correlations between TLs and APOs. Second, the GWAS dataset for TL contained 45.8% male and 54.2% female participants, which may introduce bias. Due to a lack of information for the female subgroup, we were unable to examine our results stratified according to the sex classification of TL. We anticipate that sex-disaggregated TL data will be utilized in the future to investigate the relationship between TL and APOs in greater depth. Aside from that, the majority of participants in this study were of European descent, reducing population stratification bias but limiting the applicability of our findings to other populations. Finally, Even though the F-statistic confirmed that no weak IVs existed, we observed low statistical power (below 80 percent) for certain phenotypes, which could result in false negatives.

## 5 Conclusion

Our MR study provides strong evidence that genetically predicted shortened telomeres are associated with a higher risk of SA. The potential mechanisms require further examination. UVMR and MVMR findings showed limited evidence that TL affects the risk of PTB, PE, GDM, and FGR, illustrating that the outcomes of previous observational studies may have been confounded.

## Data availability statement

The original contributions presented in the study are included in the article/**Supplementary Material**. Further inquiries can be directed to the corresponding author.

## Ethics statement

The study used summary-level data from publicly available datasets, which were not collected at the individual level. All of the participants provided written informed consent in each of the contributing studies. Therefore, ethical approval was not obtained.

## Author contributions

XH: Conceptualization, Investigation, and Writing – original draft. TW: Investigation and Resources – original draft. CL: Revising manuscript, responding to comments, and polishing language. All authors contributed to the article and approved the submitted version.
