# Supplementary figures and images for: Univariable and multivariable Mendelian randomization investigating the effects of telomere length on the risk of adverse pregnancy outcomes
Source: Front Endocrinol (Lausanne). 2023 Aug 3;14:1225600. doi: 10.3389/fendo.2023.1225600 (PMC10435990; doi:10.3389/fendo.2023.1225600)

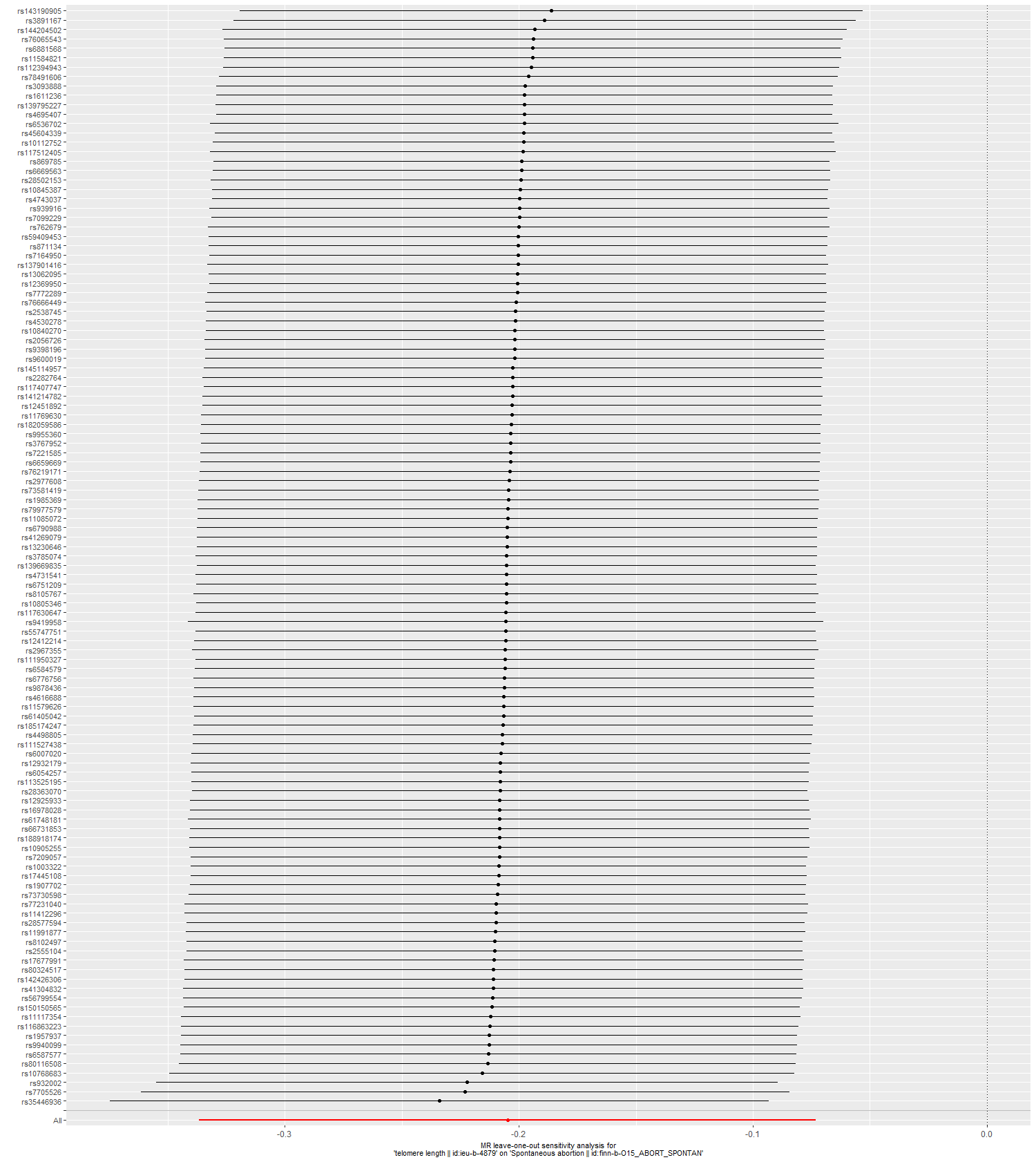

Supplement: Supplementary file 1 [file Image_1.png]

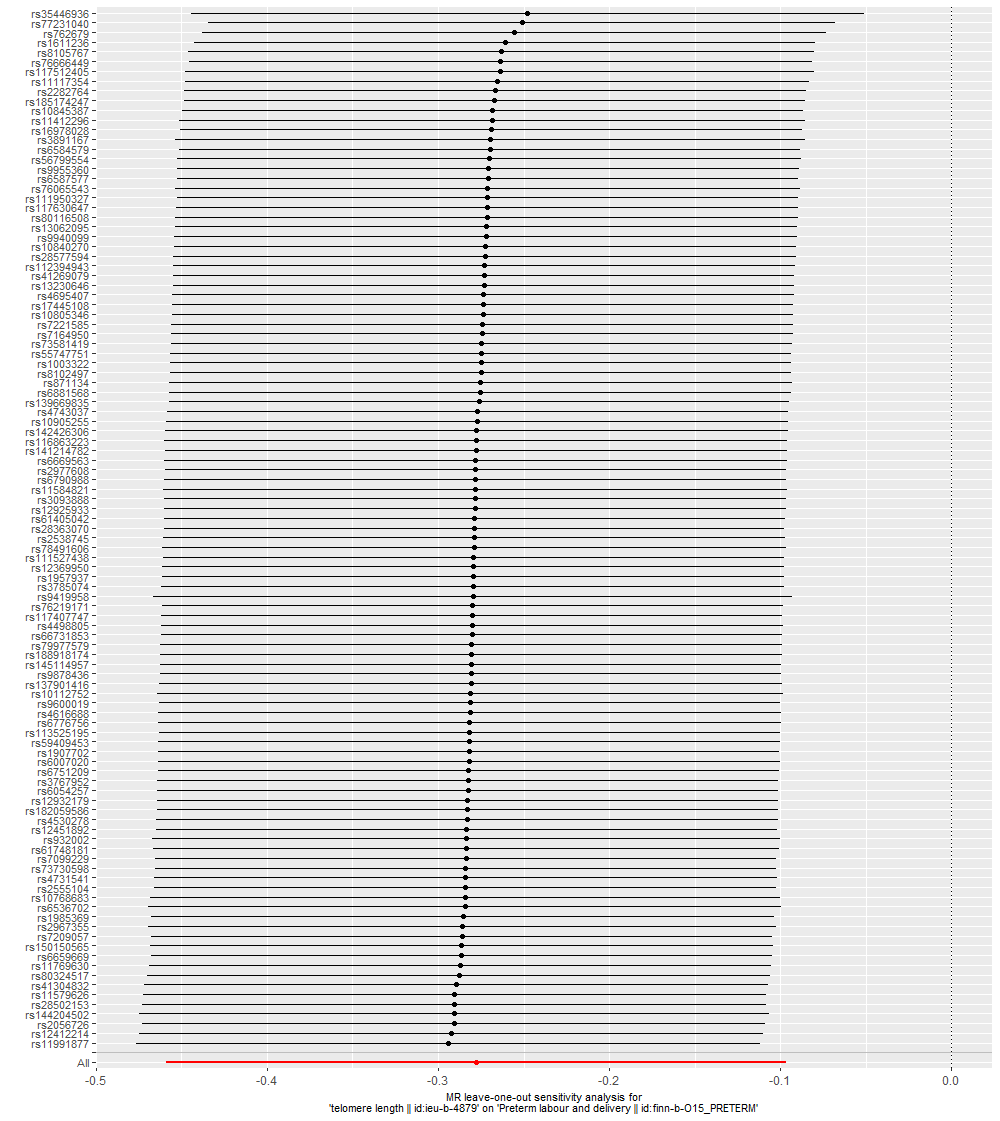

Supplement: Supplementary file 2 [file Image_2.png]

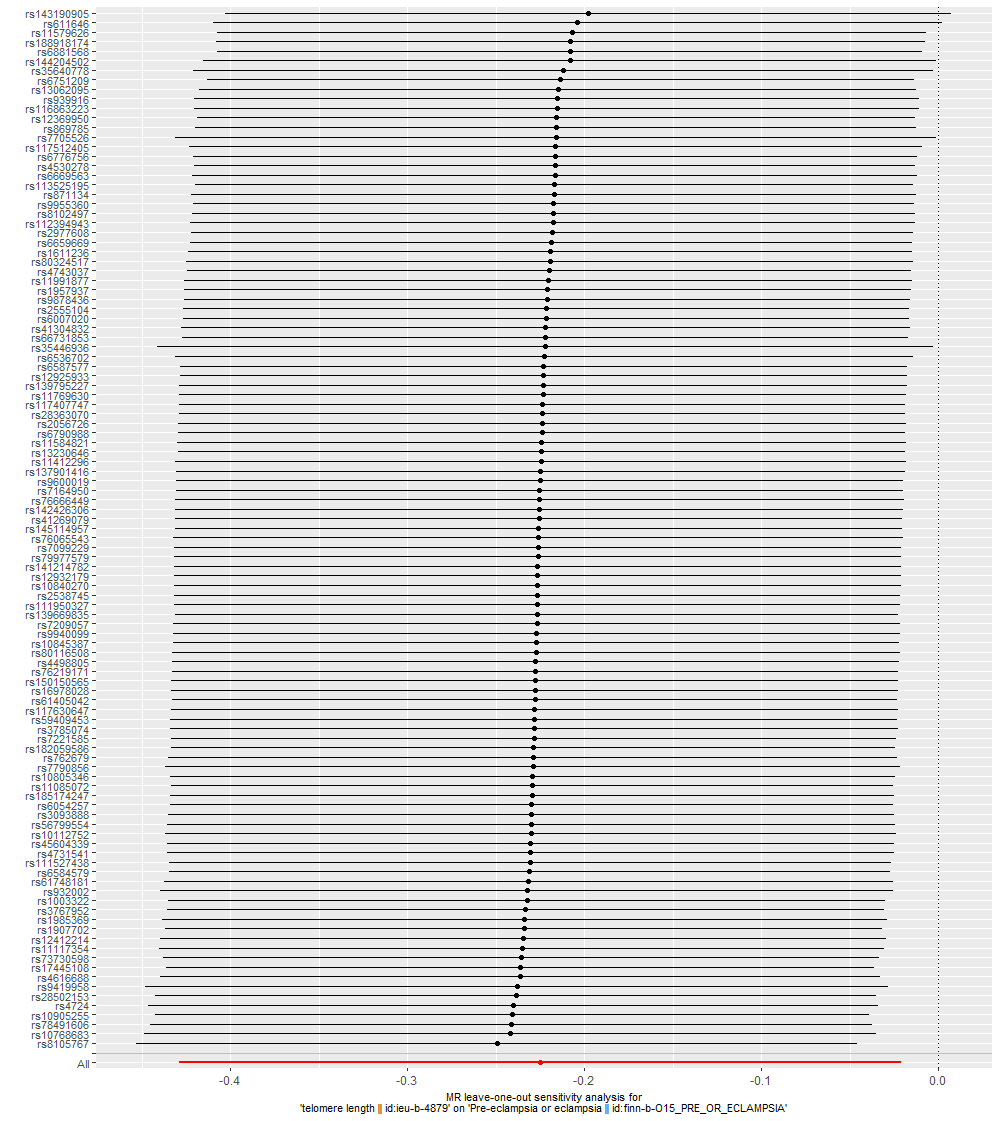

Supplement: Supplementary file 3 [file Image_3.png]

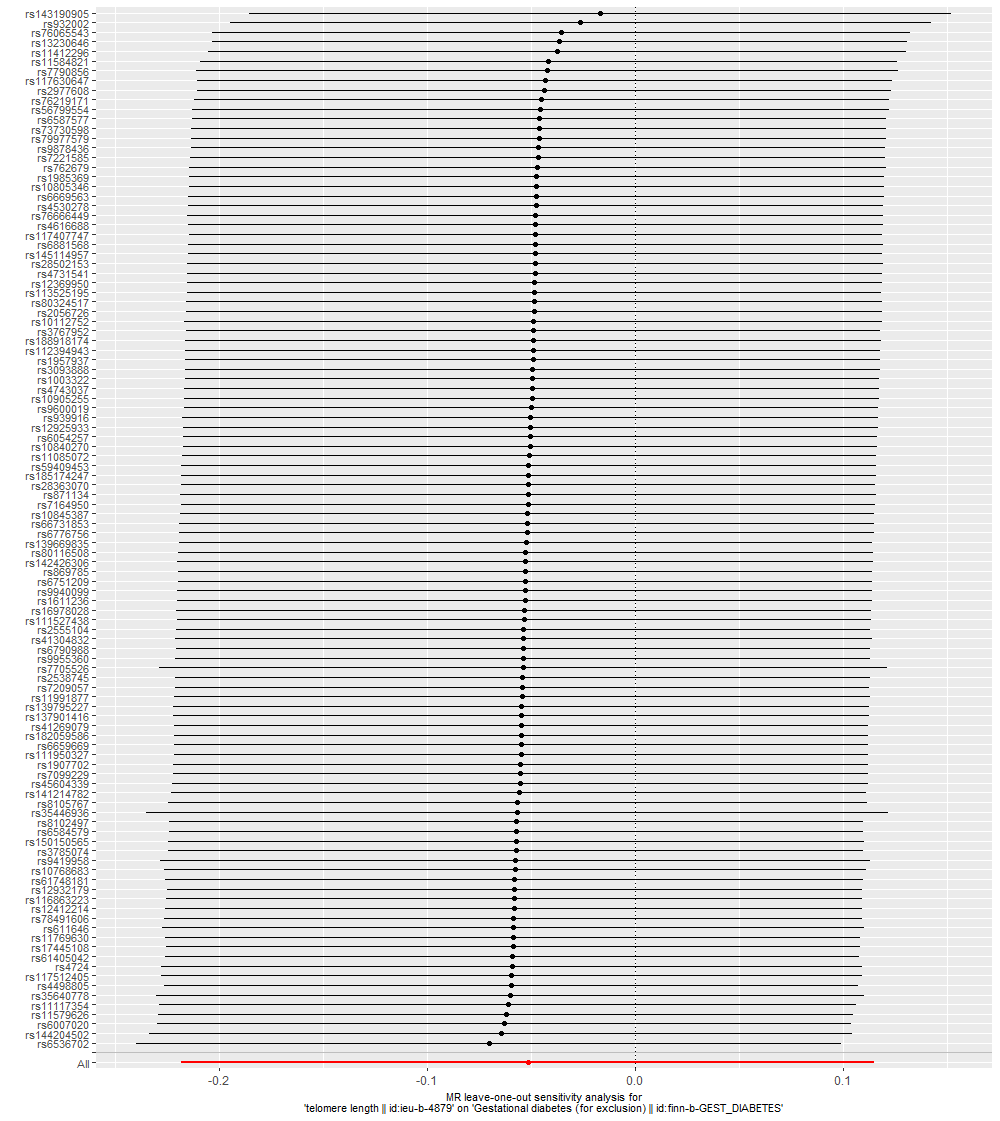

Supplement: Supplementary file 4 [file Image_4.png]

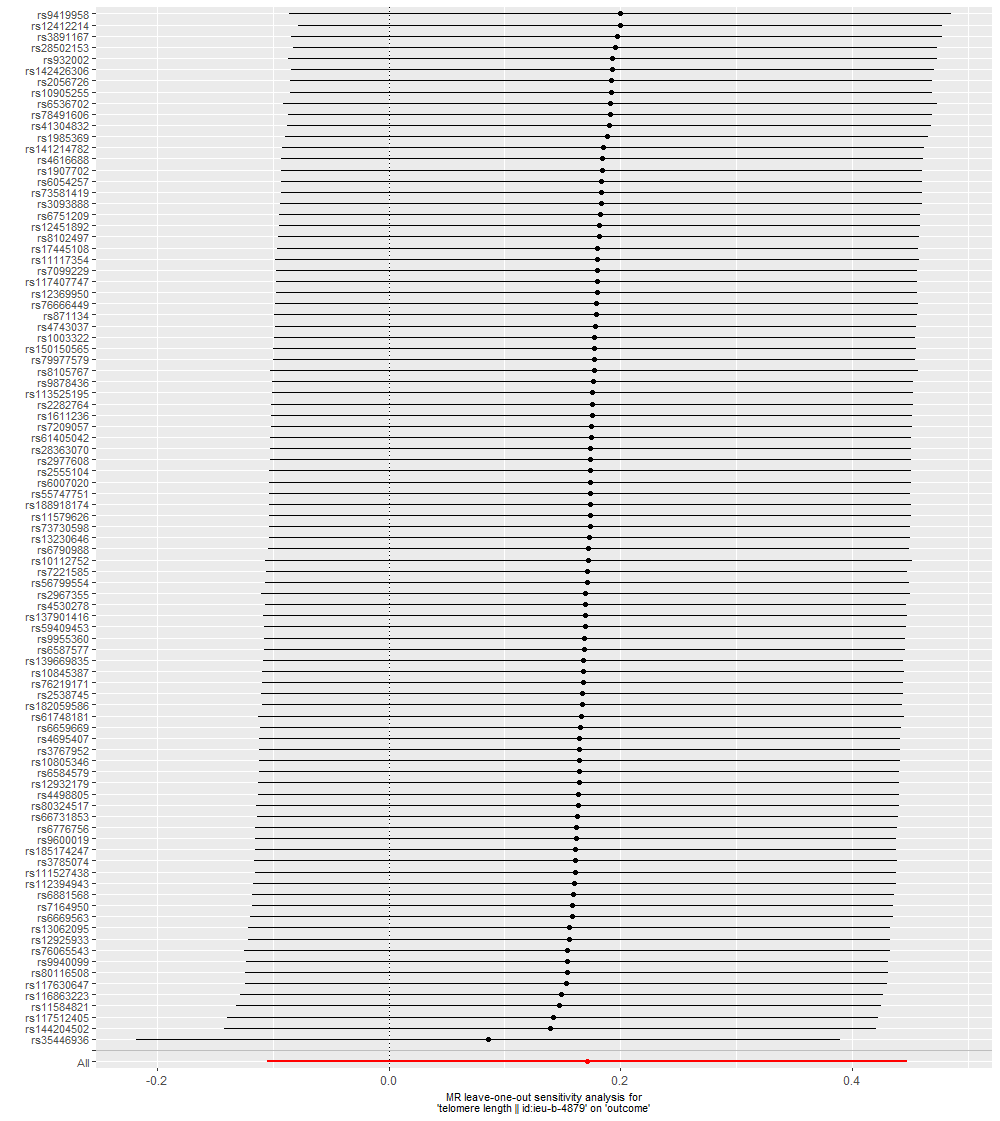

Supplement: Supplementary file 5 [file Image_5.png]

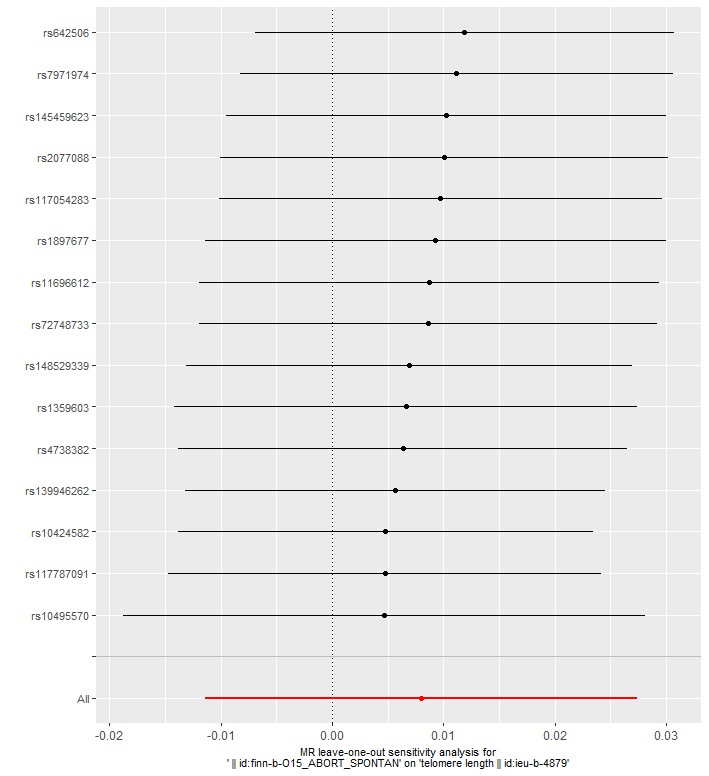

Supplement: Supplementary file 6 [file Image_6.png]

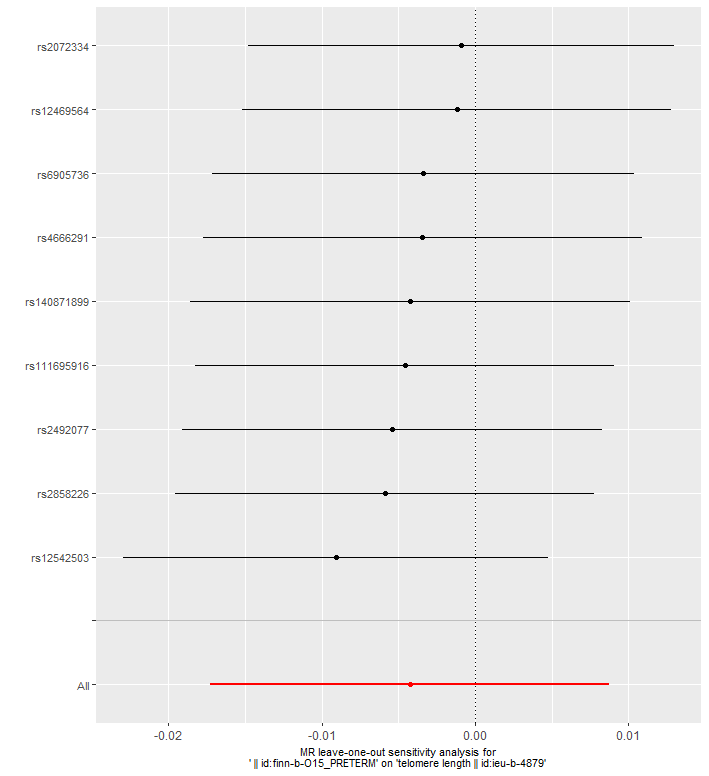

Supplement: Supplementary file 7 [file Image_7.png]

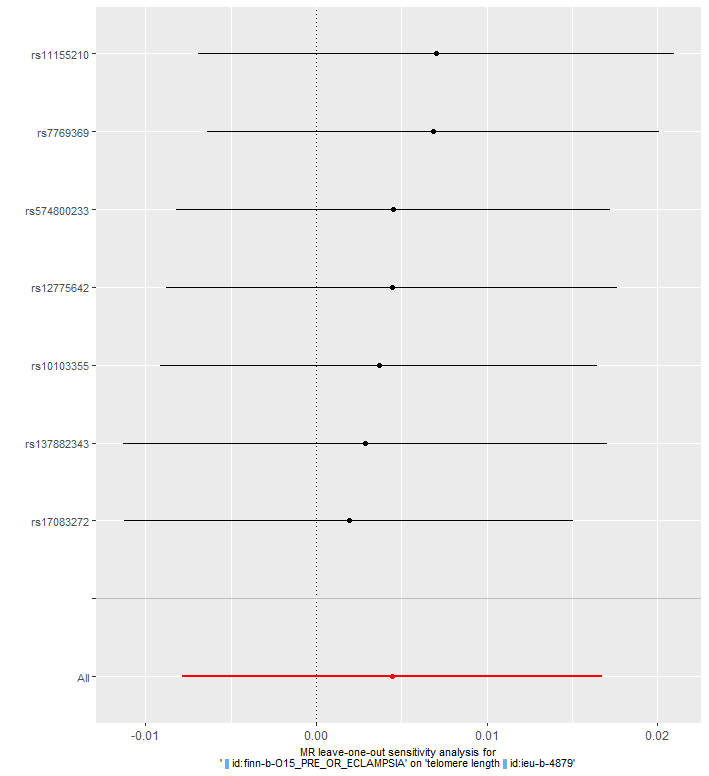

Supplement: Supplementary file 8 [file Image_8.png]

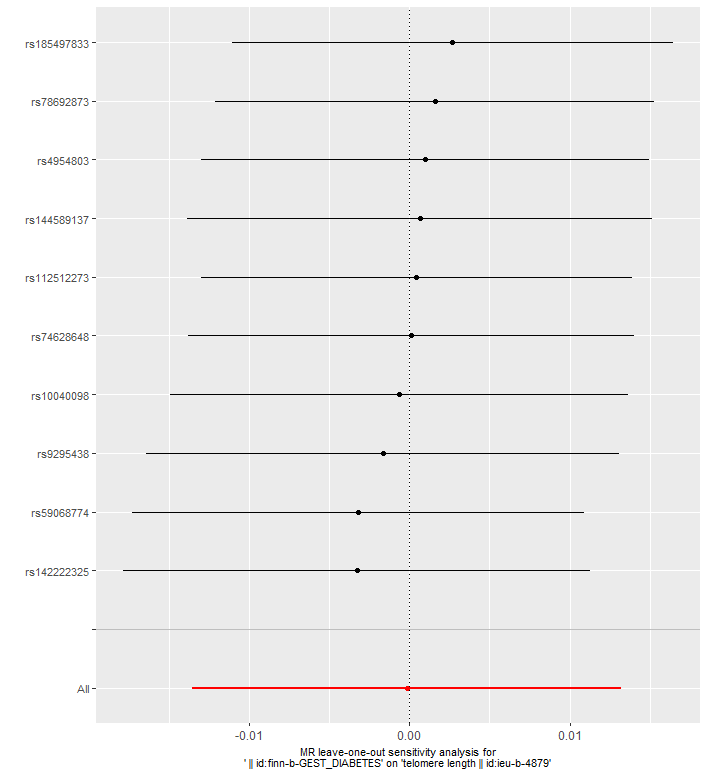

Supplement: Supplementary file 9 [file Image_9.png]

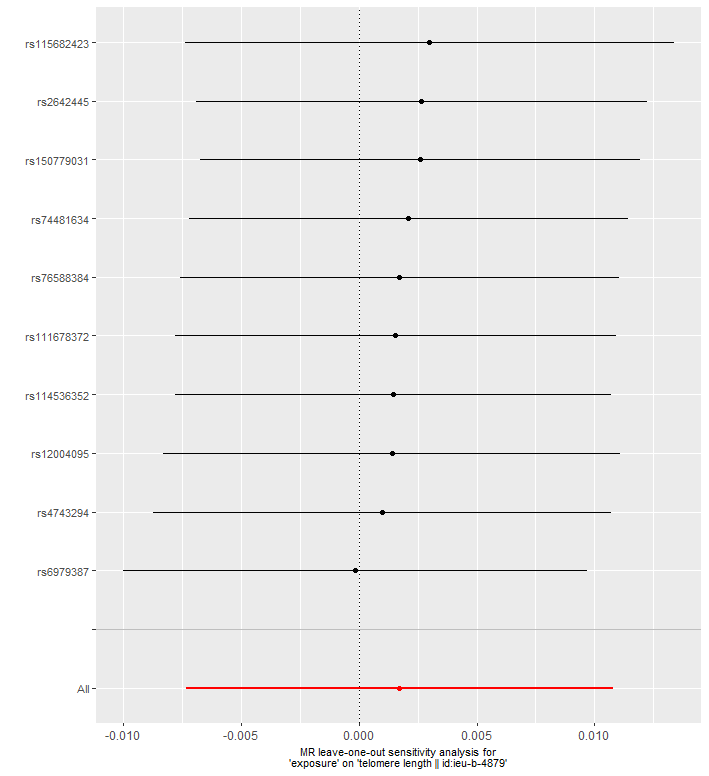

Supplement: Supplementary file 10 [file Image_10.png]

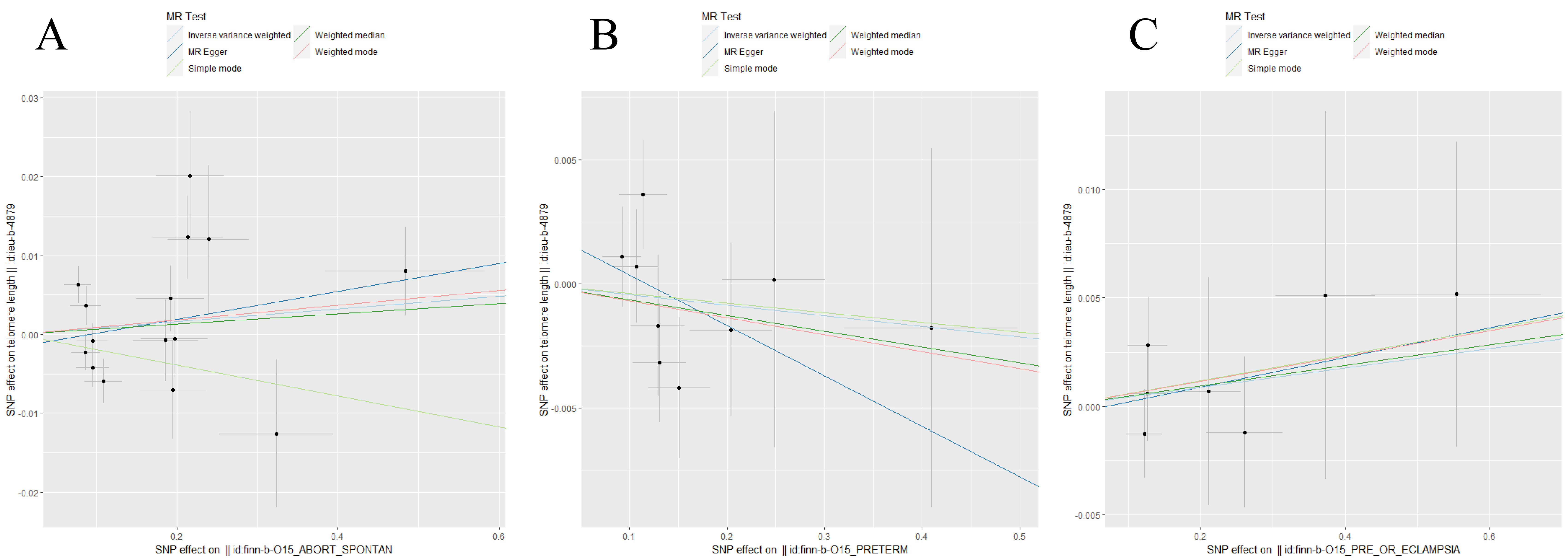

Supplement: Supplementary file 11 [file Image_11.png]

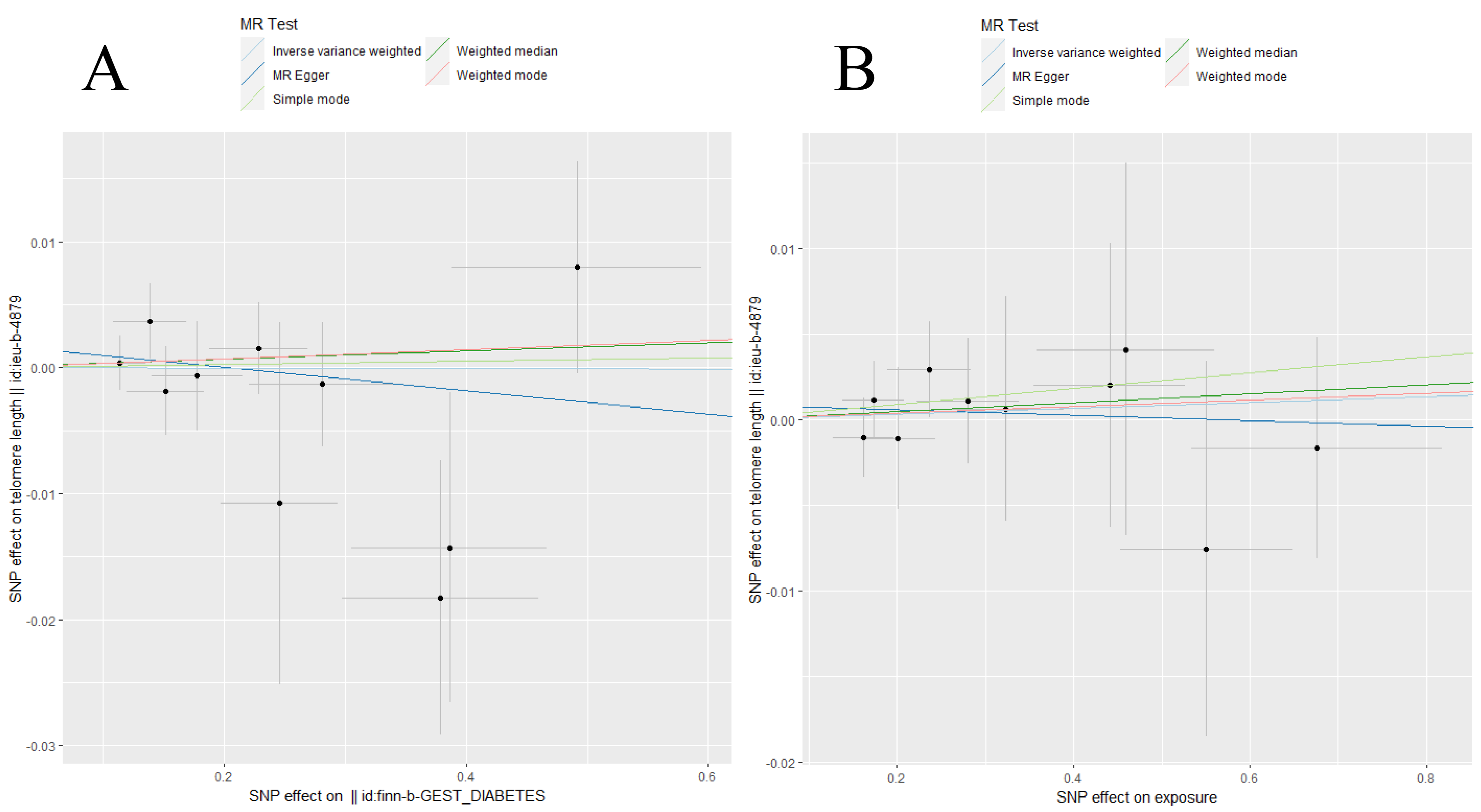

Supplement: Supplementary file 12 [file Image_12.png]
